# Molecular mechanisms of mitochondrial DNA release and activation of the cGAS-STING pathway

**DOI:** 10.1038/s12276-023-00965-7

**Published:** 2023-03-24

**Authors:** Jeonghan Kim, Ho-Shik Kim, Jay H. Chung

**Affiliations:** 1 Department of Biochemistry, The Catholic University of Korea College of Medicine, Seoul, 06591 South Korea; 2 Laboratory of Obesity and Aging Research, Cardiovascular Branch, National Heart Lung and Blood Institute, National Institutes of Health, Bethesda, MD 20892 USA

## Abstract

In addition to constituting the genetic material of an organism, DNA is a tracer for the recognition of foreign pathogens and a trigger of the innate immune system. cGAS functions as a sensor of double-stranded DNA fragments and initiates an immune response via the adaptor protein STING. The cGAS-STING pathway not only defends cells against various DNA-containing pathogens but also modulates many pathological processes caused by the immune response to the ectopic localization of self-DNA, such as cytosolic mitochondrial DNA (mtDNA) and extranuclear chromatin. In addition, macrophages can cause inflammation by forming a class of protein complexes called inflammasomes, and the activation of the NLRP3 inflammasome requires the release of oxidized mtDNA. In innate immunity related to inflammasomes, mtDNA release is mediated by macropores that are formed on the outer membrane of mitochondria via VDAC oligomerization. These macropores are specifically formed in response to mitochondrial stress and tissue damage, and the inhibition of VDAC oligomerization mitigates this inflammatory response. The rapidly expanding area of research on the mechanisms by which mtDNA is released and triggers inflammation has revealed new treatment strategies not only for inflammation but also, surprisingly, for neurodegenerative diseases such as amyotrophic lateral sclerosis.

## Inflammatory diseases: Understanding mitochondrial DNA release

Cytosolic DNA activates the cGAS-STING pathway which mediates inflammation and antiviral response. One source of cytosolic DNA is ‘self ‘ DNA, such as mitochondrial DNA. Studies of how mitochondria can release DNA and trigger dangerous immune responses are revealing potential treatments for inflammatory diseases. Cells isolate DNA in their nuclei and mitochondria, but if this ‘self-DNA’ leaks out, it triggers the same immune responses that the body uses to fight DNA from viruses or bacteria. Jeonghan Kim at The Catholic University of Korea College of Medicine in Seoul, South Korea, and co-workers reviewed research into the mechanism causing mitochondrial DNA release, and the resulting inflammatory pathways. Although cGAS-STING is the central driver of such inflammation, treatments targeting it may also suppress the body’s antimicrobial immunity. A safer alternative maybe to inhibit mitochondrial VDAC oligomerization, which releases mitochondrial DNA.

## Introduction

DNA, the blueprint of life, contains the genetic information needed for all organisms to develop, survive, and reproduce. In addition to carrying genetic information, DNA signals the host immune system to pathogens, such as DNA viruses and bacteria, inducing its response^1^. Pathogen-derived nucleic acids, such as single-stranded (ss) or double-stranded (ds) DNA and RNA, and RNA–DNA hybrids are recognized by various sensors in host cells and trigger signaling pathways to elicit innate immune responses involving the production of type I interferons (IFNs) and proinflammatory cytokines^2,3^.

Host cells initiate innate immune responses upon recognition of various types of conserved pathogen structures called pathogen-associated molecular patterns (PAMPs), such as lipopolysaccharide (LPS), yeast zymosan, and bacterial flagellin, as well as pathogen-derived nucleic acids^3^. Recent studies have indicated that damage-associated molecular patterns (DAMPs), which are endogenous factors released from damaged, dead, or transformed cells, also activate innate immune responses^3,4^. Pattern recognition receptors (PRRs) are critical for sensing both PAMPs and DAMPs in the extracellular space, endosome, and cytoplasm^3,5^. Over the over the past two decades, various classes of PRRs have been identified, including Toll-like receptors (TLRs), c-type lectin-like receptors (CLRs), retinoic acid-inducible gene I–like receptors (RLRs), nucleotide-binding oligomerization domain-like receptors (NLRs), absent in melanoma 2 (AIM2)-like receptors (ALRs), and cytosolic DNA receptors^3–5^. In infected cells, pathogen-derived nucleic acids are commonly exposed in subcellular compartments, and each type of PRR recognizes them depending on the location and polynucleotide species of the nucleic acids^3,5^. For instance, the RIG-1 in the RLR family senses 5′-tri/diphosphorylated dsRNAs, the cytosolic replication intermediates of RNA viruses^6,7^. In the TLR family, TLR-3 and TLR-7/8 detect dsRNAs and ssRNAs, respectively, and TLR-9 recognizes CpG-hypomethylated DNA in endosomes^4,7^. AIM2 in the ALR family binds to cytosolic DNA to induce the formation of the inflammasome, which consists of multiprotein complexes, in myeloid cells^5,8–11^. Subsequently, inflammasome assembly leads to the maturation of pro-interleukin-1β (IL-1β) and pro-IL-18 through the activation of the protease caspase-1^10,11^.

A number of proteins have been proposed to sense DNA in the cytosol: DNA-dependent activators of IRFs (DAI), RNA polymerase III, DExD/H box helicases (namely, DDX41), and LRR-binding FLII-interacting protein 1 (LRRFIP1)^3,12,13^. However, whether some of these proteins are DNA sensors are controversial. For example, PRRs have not been universally accepted as major DNA sensors because they recognize specific DNA sequences or function only in specific cell types^3,13^. In this review, we focus on cyclic GMP-AMP synthase (cGAS), which is a recently characterized cytosolic DNA sensor that is essential for the DNA-induced immune response, irrespective of cell type or DNA sequence^3^. Upon recognition of DNA, cGAS catalyzes the formation of cyclic guanosine monophosphate (GMP)-adenosine monophosphate (AMP) (cGAMP), which is a second messenger molecule that associates with and activates the adaptor protein stimulator of interferon genes (STING)^3,14^. Activated STING recruits TANK-binding kinase 1 (TBK1), which phosphorylates the transcription factor interferon regulatory factor 3 (IRF3)^3,14–16^. Phosphorylated IRF3 homodimerizes and then is translocated into the nucleus, where it increases the expression of type I IFNs, thereby leading to antiviral immune responses^3,14,15^. STING also upregulates inflammatory cytokines and chemokines by activating the kinase IKK, which phosphorylates and inactivates the IkB family of inhibitors of the transcription factor NF-kB^7^.

As eukaryotic cells evolved, DNA was compartmentalized into the cell nucleus and mitochondria, effectively separating the main genetic material from the cytosol^3,4^. However, DNA can be released from these organelles and accumulate in the cytosol under certain pathological conditions. Thus, the cGAS-STING pathway is activated by not only exogenous DNA derived from pathogens but also self-DNA, such as mtDNA and nuclear DNA^3,4^. In addition to their primary role of producing ATP via oxidative phosphorylation (OXPHOS), mitochondria are involved in a wide range of biological processes crucial for cell proliferation and survival^4,11,17^. Despite the symbiotic relationship between the cell and mitochondria, mtDNA is a potent stimulator of inflammatory responses when it is released into the cytosol or extracellular space^11,17^.

Much is known about how self-DNA is recognized as a DAMP by PRRs to induce the production of type I IFN and inflammatory cytokines through the cGAS-STING pathway, TLR-9, and inflammasome formation^4,5,7^. In this review, we focus on the current state of the understanding of the cGAS-STING pathway and how it mediates inflammation in response to ectopic mtDNA localization. In addition, we discuss how mtDNA is released from mitochondria and how this knowledge can be leveraged to treat diseases associated with ectopic mtDNA.

## Discovery of cGAS-STING signaling

**Fig. 1:**
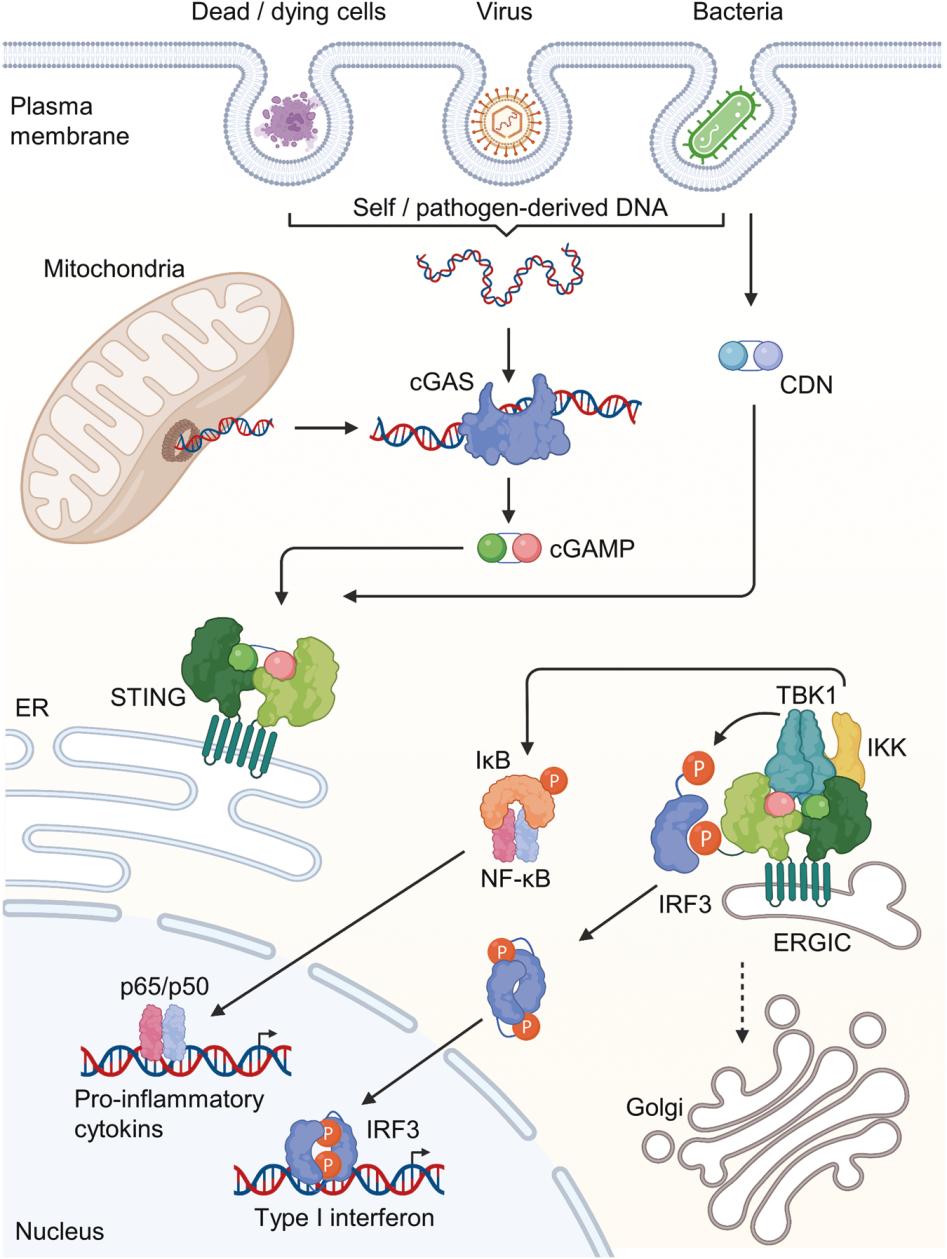
Overview of the cGAS-STING signaling pathway. The host immune response is initiated by the recognition of cytosolic DNA, such as pathogen-derived nucleic acids (e.g., viruses and bacteria) and self-DNA (e.g., from dying cells, tumor cells or mtDNA). Upon recognition of cytosolic DNA, cGAS catalyzes the formation of cGAMP and thereby activates STING. STING then activates TBK1, which phosphorylates IRF3. Phosphorylated IRF3 activates the expression of type I IFNs in the nucleus. STING also activates NF-κB by phosphorylating the kinase IKK. NF-κB then activates the transcription of genes encoding proinflammatory cytokines.

In screening performed to identify mediators of the type-I IFN response, several research groups independently identified a protein called STING that is activated by DNA and upregulates type-I IFN responses^1,18,19^. It was initially postulated that STING directly detects cytosolic DNA and triggers antiviral gene expression. Subsequently, it was found that the ligand of STING was a cyclic dinucleotide (CDN), not DNA. STING recognizes cyclic diadenosine monophosphate (c-dAMP) and cyclic diguanylate monophosphate (c-dGMP) in bacteria^20^, as well as 2’3’-cGAMP, which is produced by cGAS (also known as MB21D1 or C6orf150) upon its binding to DNA in mammalian cells^12,21–23^ (Fig. 1). STING is composed of a basic, unstructured N-terminal domain, a central nucleotidyltransferase (NTase, the catalytic sequence) domain, and a C-terminal Mab-21-homologous domain that binds to DNA via the positively charged Zn^2+^-ribbon/thumb motif^3,24^.

Although cGAS has been mostly reported to be a cytoplasmic protein, it has also been found in the nucleus and on the plasma membrane^3,15,25,26^. cGAS interacts with a viral capsid sensor in the nucleus, and it has been proposed that nuclear or perinuclear localization of cGAS facilitates viral DNA detection during the integration and replication phases of viruses^25^. cGAS has also been reported to bind to the inner leaflet of the plasma membrane through its interaction with phosphatidylinositol 4,5-bisphosphate (PI(4,5)P2)^26^. An obvious advantage of plasma membrane localization is microbe detection at the point of cell entry^26^.

Upon binding to DNA, cGAS undergoes a conformational change that leads to a rearranged catalytic pocket of the enzyme to allow ATP or GTP binding, thereby synthesizing the second messenger cGAMP^3,12,15,23^. cGAS binds cytosolic dsDNA without sequence specificity, but it binds only B-form dsDNA, not A-form dsDNA^3^. Although other types of nucleic acids, such dsRNA, can bind to cGAS, these species do not stimulate cGAS activity^3,12^. However, cGAS specificity is still controversial because some studies have suggested that cGAS senses synthetic RNA/DNA hybrids or unpaired DNA nucleotides flanking short base-paired DNA stretches derived from human immunodeficiency virus type-1 (HIV-1)^13,27^.

**Fig. 2:**
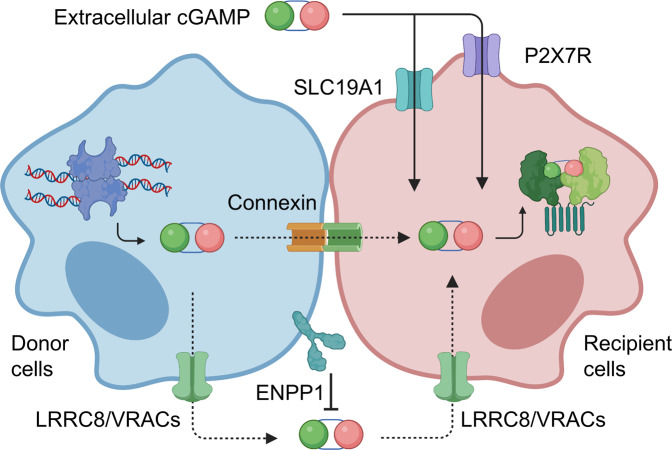
cGAS activation by DNA binding. cGAS binds to dsDNA ≥18 bp and can form dimers when the DNA sequence is sufficiently long or is bent by other factors (e.g., TFAM). Liquid phase condensation leads to the stable ladder/net interaction of cGAS and enhances its catalytic activity.

As suggested by its lack of sequence specificity, cGAS binds to DNA only on the sugar-phosphate backbone, without interacting with bases^2,3^. However, cGAS requires a minimum of 18 bp of dsDNA for binding, and dsDNA ≥40 bp can form a more stable “ladder network” with cGAS, further increasing the protein catalytic activity^2,22,28^. In addition, the presence of long DNA sequences leads to a dimeric structure (a 2:2 DNA:cGAS complex) that promotes the liquid phase condensation, further stabilizing the active dimeric state through multivalent interactions between nearby cGAS molecules^2,29,30^ (Fig. 2).

### Posttranslational modification of cGAS

A complex network of pathways modulates cGAS activity both before and after DNA binding. Protein kinase B (PKB), also known as Akt, phosphorylates human cGAS at the Ser305 residue (Ser291 in mouse cGAS) near the catalytic site of cGAS^31^, suppressing cGAMP synthesis^31^. Similarly, DNA-dependent protein kinase (DNA-PK) phosphorylates Thr68 and Ser213 and suppresses cGAMP synthesis and antiviral innate immunity^32^. When cells enter mitosis, aurora kinase B (AurB) hyperphosphorylates the N-terminus of cGAS to suppress cGAS activity, thereby preventing cGAS from sensing chromatin^33^. Protein phosphatase 6 catalytic subunit (PPP6c), which is a member of the serine/threonine protein phosphatase family, is constitutively associated with cGAS, dephosphorylating human cGAS at the Ser 435 residue (Ser420 in mouse cGAS) in resting cells^34^. This interaction prevents autoimmune reactions by retaining cGAS in the inactive state in the absence of ligands^34^. In addition to phosphorylation, glutamylation plays an essential role in the regulation of cGAS activity. The polyglutamylation of mouse cGAS at Glu272 by tubulin tyrosine ligase-like enzyme 6 (TTLL6) abolishes the cytosolic dsDNA-binding ability of cGAS, and the monoglutamylation of cGAS at Glu302 by TTLL4 suppresses cGAS catalytic activity^35^. Ubiquitination and SUMOylation are also reversible posttranslational modifications of cGAS. During the early phase of viral infection, tripartite motif-containing 38 (Trim38, an E3 ubiquitin ligase) stabilizes cGAS by preventing its Lys48 (K48)-linked ubiquitination and subsequent degradation^36^. In the late phase of a viral infection, SUMO-specific protease 2 (SENP2) deSUMOylates cGAS, leading to cGAS degradation through the ubiquitin-proteasomal or chaperone-mediated autophagy pathways^36^. Ring finger protein 185 (RNF185, an E3 ubiquitin ligase) interacts with cGAS during viral infections and specifically catalyzes the K27-linked polyubiquitination of cGAS at K137 and K384, enhancing the enzymatic activity of cGAS^37^. K48-linked ubiquitination at K414 promotes p62-mediated autophagic degradation of cGAS, whereas tripartite motif-containing 14 (Trim14) recruits ubiquitin-specific peptidase 14 (USP14) to cleave the K48-linked ubiquitin chains of cGAS at K414 and increase cGAS stability^38^.

### Intercellular cGAMP transmission and signaling networks

Interestingly, cGAMP in the extracellular space can propagate the intercellular immune response. For example, direct administration of cGAMP to mice induced an innate immune response by upregulating IFN expression, which did not occur in STING-deficient mice^39–41^. On the other hand, ecto-nucleotide pyrophosphatase phosphodiesterase 1 (ENPP1) degraded extracellular cGAMP and blunted the host immune response^42,43^. Mutating histidine 362 led to the loss of its cGAMP-degradation activity and enhanced cellular resistance to HSV-1 infection but induced systemic inflammation by enhancing STING signaling^44^.

**Fig. 3:**
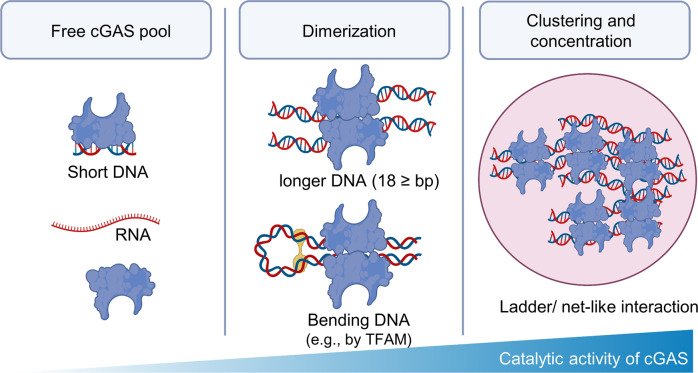
Intracellular cGAMP transmission. Intracellular cGAMP can be transferred to neighboring or adjacent cells through gap junctions, formed by the connexin protein, or via plasma-membrane transporters, including SLC19A1, P2X7R, and LRRC8. Degradation of extracellular cGAMP by ENPP1 modulates extracellular cGAMP levels.

The extracellular function of cGAMP indicates that intracellular cGAMP can be released into the extracellular space through transmembrane carriers, such as channels or extracellular vesicles, and various nonspecific biological mechanisms may induce extracellular cGAMP activation of intracellular STING. A genome-wide CRISPR led to the identification of solute carrier family 19 member 1 (SLC19A1), which can import exogenous cGAMP in a cell- and species-dependent manner^45,46^. In a subsequent study, the purinergic P2X7 receptor (P2X7R) was identified as a novel transporter of extracellular cGAMP released from membrane- compromised dying tumor cells^47–49^. Another transporter of cGAMP is leucine-rich repeat-containing protein 8 (LRRC8)A/E, a subunit of the volume-controlled anion channel (VRAC), which has been shown to mediate both the efflux and influx of cGAMP and other CDNs^49^. Indeed, genetic ablation or chemical blockade of LRRC8A/E-containing VRACs reduced the intercellular transport of cGAMP and antiviral immune response^49,50^ (Fig. 3). It is possible that extracellular cGAMP can be sensed directly. One of the three alternatively spliced isoforms of STING is localized to the plasma membrane, and the CTD of the spliced STING isoform is secreted from the cell due to the lack of a transmembrane domain, in contrast in canonical STING^51^. This isoform is expressed with the same pattern as the canonical isoform of endoplasmic reticulum STING and activates the TBK1/IRF3/IFN pathway^51^. The intercellular transfer of cGAMP may not require its release into the extracellular space. Intracellular cGAMP can be delivered via intercellular connexin proteins that form gap junctions with adjacent cells, as well as via extracellular vesicles, such as exosomes, microparticles, and viral particles^52–55^.

### Activation of STING

**Fig. 4:**
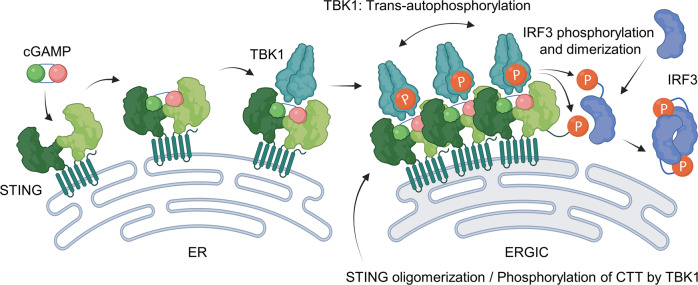
Regulation of STING and the downstream signaling pathway. Upon binding to cGAMP, STING undergoes a conformational change, and then, the dimers oligomerize. STING oligomerization recruits TBK1 and promotes autotransphosphorylation of TBK1 at the ERGIC. Activated TBK1 phosphorylates the CTT of STING to generate IRF3 docking sites and then phosphorylates the recruited IRF3.

STING (also named TMEM173, MITA, MPYA, and ERIS) is an endoplasmic reticulum (ER) membrane protein^12,15,23^. It is composed of an N-terminal transmembrane domain with four transmembrane helices, a cytosolic ligand-binding domain (LBD), and a C-terminal tail (CTT) with a phosphorylation motif and a TBK1-binding motif^56,57^. The crystal structure of STING shows that the LBD generates a V-shaped ligand pocket through dimerization with its cytoplasmic domain under steady-state conditions^58–60^. Upon binding to cGAMP, STING undergoes a substantial conformational change to tightly hold the ligand and then transforms into a tetrameric or higher-ordered oligomeric structure, which migrates to the ER–Golgi intermediate compartment (ERGIC) or the Golgi apparatus^60–62^. The oligomeric assembly recruits TBK1 to a highly conserved motif in the CTT of STING and leads to trans-phosphorylation of TBK1^14,63^. Activated TBK1 phosphorylates the CTT, which is directly adjacent to a p-Leu-x-Ile-Ser (pLxIS, where p indicates phosphorylation and x denotes any amino acid) motif^1,58,64^. This modification generates a docking site for IRF3 and recruits IRF3 for phosphorylation by TBK1^7,14,63,64^. Phosphorylated IRF3 homodimerizes and then is translocated into the nucleus to induce the expression of type I IFNs^7,63,64^ (Fig. 4).

Newly secreted IFNs bind to and activate heterodimeric receptor complexes consisting of IFNα receptor 1 (IFNAR1) and IFNAR2^65,66^. Dimerized IFNAR1 and IFNAR2 trigger the autophosphorylation of the receptor-associated protein tyrosine kinase Janus kinase 1 (JAK1), which phosphorylates and activates signal transducer and activator of transcription 1 (STAT1) and STAT2^67^. Phosphorylated STAT1 and STAT2 dimerize and then interact with IFN-regulatory factor 9 (IRF9) to form a trimolecular complex called IFN-stimulated gene factor 3 (ISGF3)^68^. ISGF3 is then translocated to the nucleus and binds to IFN-stimulated response elements (ISREs), thereby activating the transcription of IFN-stimulated genes (ISGs), which defend against infection through various mechanisms, such as by inhibiting viral replication and degrading viral nucleic acids^68,69^.

The role of STING in the host immune system is not limited to type I interferon signaling. STING also stimulates NF-κB-mediated transcription to increase the expression of inflammatory cytokines and chemokines^70–72^. The precise molecular mechanisms remain unclear, as conflicting interpretations of STING regulation of the transcriptional activity of NF-κB have been proposed^71–73^. It was first suggested that TBK1 is required for the activation of IKK, which promotes the degradation of IkB family proteins, which inhibit NF-κB^71–73^. However, subsequent studies have shown that activation of NF-κB by STING is not dependent on CTT, which is required to activate TBK^74,75^. Noncanonical NF-κB responses, such as those of p52–RELB, have also been reported to be regulated by STING^70,76^. Hence, understanding how STING activates NF-κB requires more investigation.

### Regulation of STING activity through posttranslational modifications

The most common and well-characterized posttranslational modification of STING is ubiquitination. TRIM56 was identified as an interferon-inducible E3 ubiquitin ligase that promotes the K63-linked ubiquitination of STING^77,78^. TRIM56 is upregulated by dsDNA and ubiquitinates STING at K150 77, causing STING to dimerize and recruit TBK1^77^. In contrast, RING finger 5 (RNF5, an E3 ubiquitin ligase) and RNF90 also ubiquitinate STING at K150^79,80^ but catalyze K48-linked ubiquitination of STING, which causes the degradation of STING through the proteasome-dependent pathway and thus reduces the cellular antiviral response^79,80^. Similar to TRIM56, TRIM32 catalyzes the K63-linked ubiquitination of STING at K20, 150, 224, and 236, which increases STING-induced IFN expression by recruiting TBK1 to STING^81^. Endoplasmic reticulum-localized autocrine motility factor receptor (AMFR, an E3 ubiquitin ligase) catalyzes the K27-linked polyubiquitination of STING at K137, K150, K224, and K236 in an insulin-induced gene 1 (INSIG1)-dependent manner in the presence of cytosolic DNA^82^. This modification causes STING and TBK1 to congregate at perinuclear microsomes as mediated through the Golgi apparatus and activates IRF3^82^.

The counterpart to STING in the antiviral response against dsRNA viruses is mitochondrial antiviral-signaling protein (MAVS, an antiviral adaptor protein with activity against RNA viruses), and its regulation is quite complex. In uninfected cells, MAVS is maintained in an inactivated state by RNF115, which catalyzes the K48-linked ubiquitination of MAVS^83^. After viral infection, RNF115 disassociates from MAVS and catalyzes the K63-linked ubiquitination of STING at K20, K224, and K289, thereby promoting MAVS translocation to the ERGIC and the recruitment of TBK1^83^. The E3 ubiquitin ligase RNF26 then catalyzes the K11-linked polyubiquitination of STING at K150, thereby protecting STING from K48-linked ubiquitination and degradation in the early phase of viral infection^84^. On the other hand, these modifications suppress antiviral activity by degrading IRF3 in an autophagy-dependent manner in the late phase of viral infection. Mitochondrial E3 ubiquitin-protein ligase 1 (MUL1, an E3 ubiquitin ligase) catalyzes the K63-linked ubiquitination of STING at K224, which is a required modification for IRF3 activation but not for NF-κB activation^85^. The K6-linked ubiquitination of STING at K19 by TRIM13, K48-linked ubiquitination of STING at K83, K151, K288, and K337 by TRIM29, and K48-linked ubiquitination of STING at K275 by TRIM30α promote proteasome-dependent STING degradation, thereby suppressing antiviral responses^86–88^. Thus, K11-, K27-, or K63-linked ubiquitination mostly enhances the STING-mediated antiviral response, whereas K6- or K48-linked ubiquitination suppresses this response.

### The cause and immunostimulatory effect of mtDNA release

The endosymbiotic model, which indicates that mitochondria are derived from ancient aerobic bacteria, mitochondria and bacteria share several similar features, such as a circular genome and hypomethylated/unmethylated CpG dinucleotide motifs^89^. Owing to these similarities, mitochondrial components, such as mtDNA, are recognized as DAMPs by the host immune system upon release from mitochondria into the cytoplasmic or extracellular milieu. Recent extensive studies have shown that mtDNA is released under pathological conditions, such as oxidative stress, genotoxic stress, high levels of proinflammatory factors, viral infection, and mitochondrial dysfunction, subsequently activating the cGAS-STING pathway.

### Oxidative stress

Numerous studies have shown that oxidative stress caused by excess reactive oxygen species (ROS) production is a major source of DNA damage^4^. Intracellular ROS are predominantly produced in the electron transport chain (ETC) in the mitochondrial matrix during oxidative phosphorylation^4^. Compared to nuclear DNA, mtDNA is more vulnerable to oxidative damage because mtDNA resides near the ETC and is not protected by histones^4,10,11^. As a result, mitochondrial oxidative stress disrupts mtDNA integrity, facilitating the release of oxidized mtDNA into the cytoplasm and eventually into the extracellular space, thereby triggering IFN and proinflammatory responses^4,10,11,17^.

Three prime exonuclease 1 (TREX1), which is localized to the ER in the basal state, degrades cytosolic DNA and prevents the activation of the cGAS-STING pathway^90–92^. Loss of TREX1 activity in humans and mice causes autoimmune diseases such as systemic lupus erythematosus (SLE) and Aicardi–Goutieres syndrome^91,92^. Oxidized DNA is resistant to TREX1-mediated degradation and therefore accumulates in the cytosol, leading to cGAS activation^90^. Therefore, one can infer that mtDNA released into the cytosol or extracellular space in response to oxidative damage induces particularly robust inflammatory responses. Indeed, in SLE, oxidized mtDNA-protein complexes are released from mitochondria into the cytoplasm in the neutrophils and are spontaneously extruded to the extracellular space^93^. The extrusion of oxidized mtDNA in turn activates plasmacytoid DCs and causes the production of anti–oxidizing mtDNA autoantibodies^93^. In a mouse model of SLE, mitochondrial oxidation induced neutrophil extracellular trap (NETosis), releasing oxidized mtDNA into the extracellular environment^94^. Consistent with this outcome, reducing mtROS via the mitochondrion-targeted antioxidant Mito-TEMPO ameliorated disease in a mouse model of SLE^94^.

### Inflammasome activation

Oxidative damage to both mitochondria and mtDNA is essential for the activation of the NOD-, LRR- and pyrin domain-containing protein 3 (NLRP3) inflammasome, which triggers IL-1β secretion from macrophages^10,95^. Activation of the NLRP3 inflammasome requires a priming step stimulated by PAMPs (e.g., LPS) or inflammatory cytokines, followed by an activation step mediated through the assembly of components, such as the NLRP3 inflammasome, apoptosis-associated speck-like protein containing a CARD (ASC), and pro-caspase-1^10,11,95^. The priming step increases the expression of inflammasome components, lowers mitochondrial membrane potential and increases mtROS levels and de novo synthesis of mtDNA^10,11,95^. Depletion of mtDNA with ethidium bromide treatment or knockdown of mtDNA polymerase-γ (POLγ) inhibited NLRP3 inflammasome activation in mouse bone marrow-derived macrophages (BMDMs)^11^. Deficient mitochondrial transcription factor A (TFAM), which regulates the replication and compaction of mtDNA, markedly reduced the mtDNA content and suppressed inflammasome activation in BMDMs^11^. TFAM ablation reduced both mtROS and ox-mtDNA in response to NLRP3 activators, although the expression levels of general inflammasome components, such as pro–IL-1β, pro-caspase1, and NLRP3, remained unchanged^11^. Treatment of TFAM-deficient BMDMs with synthetic mtDNA fragments containing the oxidized nucleotide 8-OH-dGTP restored NLRP3 inflammasome activation, supporting the notion that mtDNA synthesis during the priming step is essential for inflammasome activation^11^. Indeed, LPS promotes mtDNA synthesis by activating mitochondrial deoxyribonucleotide kinase UMP-CMPK (CMPK2), which provides dCTP for mtDNA replication and increases mtROS levels^11^. The IL-1β and IFN pathways are interlinked as indicated by IL-1β induction of IFN production and suppression of viral replication^96,97^. IL-1β also triggers mtDNA release and activates the cGAS-STING pathway by reducing mitochondrial membrane potential and increasing mitochondrial mass^97^. Thus, mtDNA release and IL-1β secretion promote chronic inflammation by driving a feed-forward loop.

### Disruption of mitochondrial integrity

Oxidatively damaged mitochondria contain small mtDNA fragments that are released. When the permeability of mitochondrial membranes was increased^98,99^, TFAM protected mtDNA from damage, but surprisingly, Tfam ± cells released more mtDNA fragments into the cytosol and exhibited higher cGAS-STING activity, as evidenced by the upregulation of ISG expression and antiviral response^17^. On the other hand, mitochondrial fission, which clears damaged mitochondria via mitophagy, suppressed the upregulation of ISG expression, but depletion of the mtDNA quality-control enzyme endonuclease G-like 1 (EXOG) increased ISG expression in these cells^17^.

YME1L deficiency also induces mtDNA release into the cytosol and triggers the innate immune response through the cGAS–STING pathway^100^. YME1L is an ATP-dependent metalloprotease located on the inner mitochondrial membrane and coordinates mitochondrial biogenesis and dynamics by balancing the fusion and fission of mitochondria^100^. YME1L deficiency causes the accumulation of numerous YME1L substrate proteins associated with mtDNA metabolism, including solute carrier family 25 member 33 (SLC25A33), CMPK2, and nucleoside diphosphate kinase (NME4)^100^. More direct evidence for the role of oxidative damage in mtDNA release has been demonstrated by studies showing the role of thioredoxin-2 (Trx2), which scavenges mtROS, in regulating mtDNA release^101^. Trx2 deficiency increased the level of mitochondrial superoxide, hydrogen peroxide and cytosolic mtDNA in brown adipose tissue^101^. Increased levels of NLRP3, cleaved caspase-1, and mature IL-1β in these cells suggested NLRP3 inflammasome activation, which, as we described above, required the release of oxidized mtDNA^101^.

#### Channels on the mitochondrial membrane involved in innate immunity

Early understanding of mtDNA release came from studying the mitochondrial apoptosis pathway, which requires mitochondrial outer membrane permeabilization (MOMP) to release cytochrome C, which initiates the apoptotic cascade^102^. The Bcl-2 family of proteins, BCL2 antagonist/killer (BAK) and BCL2-associated X (BAX), which accumulate in the mitochondrial outer membrane (MOM) in response to apoptotic stimuli, were the first mediators of MOMP to be identified^103,104^. Subsequent studies demonstrated that voltage-dependent anion channels (VDACs) were required for BAX/BAK-mediated MOMP and apoptosis^104^. At the time of its discovery, it was thought that VDAC was a component of the mitochondrial permeability transition pore (mPTP) in the mitochondrial inner membrane, the opening of which leads to mitochondrial swelling and rupture. However, VDAC has since been shown to not be a component of the mPTP^99^, raising the question, how is VDAC involved in MOMP?

#### mtDNA efflux through BAK/BAX macropores

**Fig. 5:**
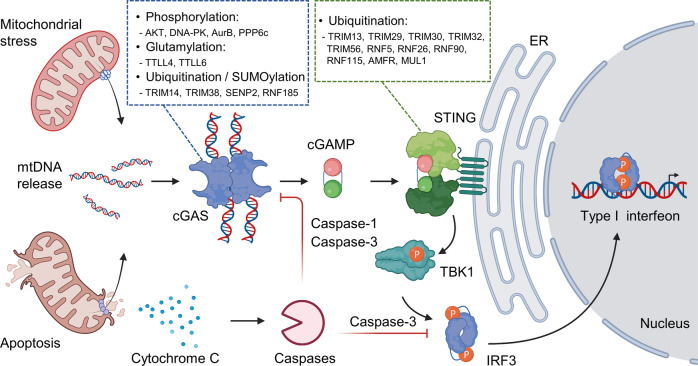
Posttranslational modification in the cGAS-STING pathway. Mitochondrial stress induces the release of mtDNA and subsequently activates the cGAS-STING pathway. This signaling pathway can be regulated by various posttranslational modifications and can also be suppressed by caspases during apoptosis.

During apoptosis, cytochrome c is released from mitochondria through the BAK/BAX pore and binds to apoptosis protease activator-1 (Apaf-1) and pro-caspase-9 to induce the apoptosome complex^105^. The assembly of apoptosomes activates caspase-9, an apoptosis-initiating caspase, which in turn activates the effector caspase-3 or caspase-7, leading to the biochemical and morphological features of apoptosis^105^. However, immunogenic mitochondrial components (e.g., mtDNA) are released during apoptosis, raising the possibility that apoptosis can trigger systemic inflammation^102,105^. Such harmful outcomes are averted by a several built-in safety mechanism. For example, infection by thrombocytopenia syndrome virus (SFTSV) activates BAK/BAX and the NLRP3 inflammasome^106^. Activation of BAK/BAX increases +intracellular [AMP]/[ATP] ratio, which activates AMPK and induces autophagy in an ATG5/7-dependent manner^107^, thereby removing damaged mitochondria, which are likely to be sources of released mtDNA. Moreover, caspase-3 is activated during apoptosis and cleaves cGAS at D319 and IRF-3 at D121/125^102,108,109^, and caspase-1 cleaves cGAS at D140/D157, further inactivating the type I IFN pathway^9^ (Fig. 5).

**Fig. 6:**
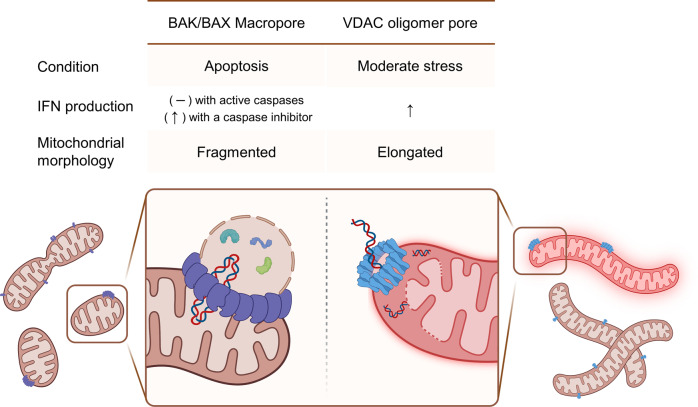
mtDNA release via permeabilization of the mitochondrial outer membrane. (Left) During BAK/BAX-mediated apoptosis, mitochondria undergo hyperfragmentation, and mitochondrial matrix components, including mtDNA, are released into the cytosol through BAK/BAX macropores. mPTP opening is not required for MIM disruption. The released mtDNA does not activate the type-I IFN pathway because of caspase activation. (Right) Mitochondrial stress triggers the formation of VDAC-oligomer pores and activates the type-I IFN pathway. mPTP opening is required for MIM disruption.

Microscopy studies of MOMP after treatment with ABT-737, a BAK/BAX activator, showed that the elongated mitochondrial network becomes hyperfragmented during apoptosis and then, BAK/BAX is oligomerized on the MOM, forming macropores^109,110^. The mitochondrial inner membrane (MIM) then herniates through the BAK/BAX macropores, releasing mitochondrial matrix components, including mtDNA, into the cytosol^109,110^ (Fig. 6).

### mtDNA release through VDAC oligomer pores

VDAC is the most abundant MOM protein and is the primary channel through which Ca^2+^, anions, cations, ATP, and metabolites are transported between mitochondria and other compartments of a cell^111^. VDAC is a β-barrel protein expressed as three isoforms (VDAC1, VDAC2 and VDAC3), of which VDAC1 is most highly and ubiquitously expressed^16,99^. Although VDACs are monomers involved in channel functions, they exist in dynamic equilibrium with the oligomeric forms^112^. Equilibrium shifts towards the oligomeric form during apoptosis, which, as mentioned above, is accompanied by MOMP and cytochrome C release^113^. Although the structure of VDAC oligomers in apoptotic cells is not known, it is reasonable to propose that they form macropores similar to BAK/BAX. All VDACs contain a flexible amphipathic N-terminal domain that lies inside the channel but is thought to translocate out of the pore when voltage is applied across the VDAC channel 98 or during apoptosis^16^. Evidence suggests that the translocated N-terminal domain may generate a hydrophilic surface around the circumference of the VDAC macropore, stabilizing the VDAC–water interface^98^. The crucial difference between VDAC and BAK/BAX pores is that VDAC oligomers can also form in living (not apoptotic) cells and require mitochondrial permeability transition pore (mPTP) opening for permeabilization of the MIM^98^. Thus, even in normally growing cells, VDAC oligomers form on stressed mitochondria, which in combination with the opening of the mPTP releases mtDNA^98^. Because these oligomers form in BAK/BAX-deficient cells, the BAK/BAX macropore is not required for MOMP in living cells^98^. Because mtDNA is released in the absence of apoptosis or caspase activity, the cGAS-STING signal remains intact and activates the type I IFN pathway.

Consistent with the notion that VDAC oligomers release mtDNA, VBIT-4, a small- molecule inhibitor of VDAC, inhibits mtDNA release and type-I IFN signaling, ameliorating inflammatory diseases such as SLE in a mouse model 98. The requisite release of mtDNA for the NLRP3 inflammasome activation has been shown to be mediated by VDAC oligomerization and blocked by VBIT-4^95^. More recently, it was discovered that COVID-19 caused type-I IFN pathology in endothelial cells by inducing VDAC oligomerization, which led to released mtDNA and activation of the cGAS-STING pathway^114^ (Fig. 6).

The pathological role of VDAC oligomerization-induced release of mtDNA extends beyond conditions classically considered to be immunity-released diseases. Amyotrophic lateral sclerosis (ALS) is a motor neuron disease that leads to paralysis and nearly 100% mortality^115^. One of the etiologies of ALS is mislocalization of TAR DNA-binding protein 43 (TDP-43) to mitochondria, causing neurotoxicity^115^. TDP-43–mediated inflammation depends on cGAS/STING, which is activated by released mtDNA^115^. TDP-43 does not induce apoptosis, and BAK/BAX are not required for TDP-43-dependent mtDNA release or inflammatory cytokine gene expression^115^. VBIT-4 or genetic deletion of VDAC1 decreases cytoplasmic mtDNA levels and inflammation in motor neurons derived from iPSCs of ALS patients and MEFs, and blockade of the cGAS/STING pathway ameliorates the related neurodegeneration^115^.

### Conclusions and perspectives

The discovery of the cGAS-STING pathway has led to a paradigm shift in which ectopically localized self-DNA, similar to microbial DNA, causes disease. In this review, we highlight the biological properties of mtDNA released into the cytosol and the mechanism by which mtDNA crosses the MOM. In addition, we presented comprehensive updates on the regulation of the cGAS-STING pathway. The mechanism by which MIM permeabilization (MIMP) occurs is not understood as well as that of MOMP. In the context of MOMP induced by the BAK/BAX activator ABT-737, MIM and the matrix herniate and rupture without opening the mPTP. On the other hand, VDAC oligomer-induced MOMP requires mPTP opening, suggesting that an influx of small molecules and water through the mPTP into the matrix may mediate MIM disruption. The requirement for mPTP opening for VDAC oligomer-mediated mtDNA release may be an indication that the VDAC macropore is much smaller than the BAK/BAX macropore and may require an outward force applied to the MIM to drive mtDNA release. Thus, inhibitors of VDAC oligomerization, along with inhibitors of the cGAS-STING pathway, may become valuable tools in treating inflammatory diseases. One potential benefit of VDAC oligomerization inhibitors in the treatment of inflammation and autoimmunity is that this strategy of targeting mtDNA release may not diminish antimicrobial immunity to the same extent as the use of anti-cGAS-STING inhibitors. Understanding how the VDAC oligomer forms and the nature of its interactions with other MOM proteins, such as Bcl-2 family members (e.g., BAK and BAX), may provide further insights for targeting MOMP as a therapeutic strategy.
